# Reassortant Clade 2.3.4.4 of Highly Pathogenic Avian Influenza A(H5N6) Virus, Taiwan, 2017

**DOI:** 10.3201/eid2406.172071

**Published:** 2018-06

**Authors:** Li-Hsuan Chen, Dong-Hun Lee, Yu-Pin Liu, Wan-Chen Li, David E. Swayne, Jen-Chieh Chang, Yen-Ping Chen, Fan Lee, Wen-Jane Tu, Yu-Ju Lin

**Affiliations:** Council of Agriculture, New Taipei City, Taiwan (L.-H. Chen, Y.-P. Liu, W.-C. Li, J.-C. Chang, Y.-P. Chen, F. Lee, W.-J. Tu, Y.-J. Lin);; US Department of Agriculture, Athens, Georgia, USA (D.-H. Lee, D.E. Swayne)

**Keywords:** highly pathogenic avian influenza virus, H5N6, Taiwan, phylogenetic analysis, clade 2.3.4.4, zoonoses, viruses, influenza

## Abstract

A highly pathogenic avian influenza A(H5N6) virus of clade 2.3.4.4 was detected in a domestic duck found dead in Taiwan during February 2017. The endemic situation and continued evolution of various reassortant highly pathogenic avian influenza viruses in Taiwan warrant concern about further reassortment and a fifth wave of intercontinental spread.

Since 1996, H5 A/goose/Guangdong/1/1996 (Gs/GD) lineage of highly pathogenic avian influenza viruses (HPAIVs) originating in Asia have caused outbreaks in Asia, Europe, Africa, and North America (*1*). The H5N1 Gs/GD lineage of HPAIV has evolved into 10 genetically distinct virus clades (0–9) and multiple subclades, including novel H5 clade 2.3.4.4 viruses, which emerged in China (*2*) and have evolved into 4 distinct genetic groups (2.3.4.4A–D) (*3*). Four 4 intercontinental waves of Gs/GD lineage HPAIV transmission have occurred: clade 2.2 H5N1 in 2005, clade 2.3.2.1c H5N1 in 2009, clade 2.3.4.4A H5Nx in 2014, and clade 2.3.4.4B H5Nx in 2016 (*4*). The clade 2.3.4.4A and B H5N8 viruses spread intercontinentally; clade 2.3.4.4A caused outbreaks in Asia, Europe, and North America during 2014–2015, and clade 2.3.4.4B H5N8 caused outbreaks in Asia, Europe, and Africa during 2016–2017 (*1*,*5*). In fall 2016, clade 2.3.4.4C H5N6 viruses caused outbreaks in South Korea and Japan (*6*). Six distinct genotypes of clade 2.3.4.4C H5N6 viruses (designated as C1–C6) were identified in South Korea and Japan during these outbreaks; these genotypes contain different polymerase acidic and nonstructural genes from low pathogenicity influenza viruses from Eurasia (*7*,*8*).

We report HPAIV H5N6 detection from a meat-type duck in Taiwan in February 2017. One dead young Pekin-type domestic duck was found on a country road near the Xiuguluan River in Hualien County during wild bird and habitat surveillance for HPAIV by the Wild Bird Society of Taipei; the carcass was forwarded to the national laboratory of the Animal Health Research Institute (Technical Appendix 1 Figure 1). We conducted complete genome sequencing and comparative phylogenetic analysis of the detected virus, A/duck/Taiwan/1702004/2017(H5N6) (Dk/Tw/17), to trace the origin and understand its genetic features.

We detected Dk/Tw/17 virus by using reverse transcription PCR and isolated the virus by using egg inoculation as described previously (*9*). We conducted an intravenous pathogenicity index test according to the World Organisation for Animal Health Manual of Diagnostic Tests and Vaccines for Terrestrial Animals (http://www.oie.int/en/international-standard-setting/terrestrial-manual). We performed full-length genome sequencing by using reverse transcription PCR amplification and Sanger sequencing (*9*). We estimated maximum-likelihood phylogenies by using RAxML (*10*) and constructed a median-joining phylogenetic network of the hemagglutinin gene by using NETWORK 5.0 (Technical Appendix 2).

We classified Dk/Tw/17 as an HPAIV on the basis of the amino acid sequence at the hemagglutinin cleavage site (PLRERRRKR/G) and its high lethality in chickens (intravenous pathogenicity index 3.0). Necropsy and histologic examination revealed virus-specific necrotic and inflammatory lesions in the pancreas, heart, and brain (Technical Appendix 1 Figure 2). Phylogenetic analyses suggested that the Dk/Tw/17 virus belongs to clade 2.3.4.4C genotype C5 that was found in China, South Korea, and Japan during 2016–2017 (Figure, panel A; Technical Appendix 1 Figure 1). This virus genotype acquired its polymerase acidic gene of low pathogenicity influenza viruses from Eurasia; its other genes originated in the G1.1.9 and G1.1-like lineages of H5N6 viruses from China (*7*,*8*). All 8 gene segments shared high levels of nucleotide identity (99.3%–99.9%) with H5N6 viruses identified from wild birds in Japan and South Korea in November 2016, including A/whooper swan/Korea/Gangjin 49-1/2016 (H5N6), A/spot billed duck/Korea/WB141/2016 (H5N6), and A/teal/Tottori/2/2016 (H5N6) (Technical Appendix 1 Table). These viruses consistently clustered together with high bootstrap value (>70) in maximum-likelihood phylogenies across all 8 gene segments (Technical Appendix 1 Figures 3–10).

**Figure:**
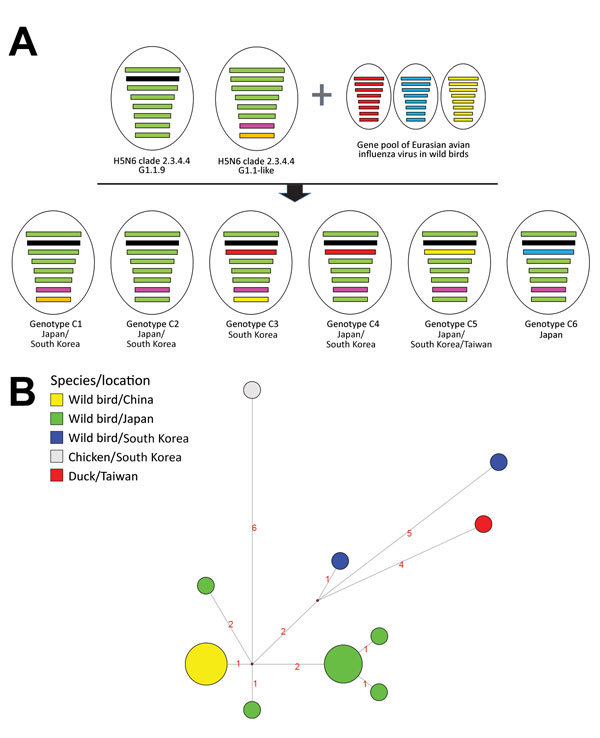
Genome constellation of influenza A(H5N6) viruses identified in East Asia during 2016–2017 and median-joining phylogenetic network of genotype C5. A) Viruses are represented by ovals containing horizontal bars that represent 8 gene segments (top to bottom: polymerase basic 2, polymerase basic 1, polymerase acidic, hemagglutinin, nucleoprotein, neuraminidase, matrix, and nonstructural). The colors of gene segments denote the genetic origins as previously described by Takemae et al. (*7*): green and black, G1.1.9 genotypes from China; pink and orange, G1.1-like genotypes from China; other colors, avian influenza lineages from Eurasia. B) Median-joining phylogenetic network of genotype C5 was constructed from the hemagglutinin gene and includes all the most parsimonious trees linking the sequences. Each unique sequence is represented by a circle sized relative to its frequency in the dataset. Branch length is proportional to the number of mutations. Isolates are colored according to the origin of the sample.

The genotype C5 comprises 17 H5N6 HPAIVs identified from wild waterfowl in China, Japan, and South Korea during November–December 2016; a virus identified from a chicken farm (A/chicken/Korea/H23/2016 [H5N6]) in South Korea in November 2016; and the Dk/Tw/17 virus. Genotype C5 is phylogenetically distinct from viruses that caused outbreaks in poultry farms in Japan and South Korea during 2016–2017. This genotype has independently evolved and been maintained in wild bird populations in the bird flyway of East Asia, highlighting how wild waterfowl play an important role in the maintenance and dissemination of this HPAIV. In addition, the median-joining phylogenetic network analysis suggests that the A/chicken/Korea/H23/2016 (H5N6) is not the direct ancestor of the Dk/Tw/17 virus, which was likely caused by separate introduction from wild birds (Figure, panel B).

The site where the dead duck was collected is adjacent to a river and located near many ponds used for duck farming. After identification of Dk/Tw/17, intensified active surveillance conducted over 3 months detected additional clade 2.3.4.4C H5N6 HPAIVs from 12 farms in 4 counties (Technical Appendix 1 Figure 1). Clade 2.3.4.4A H5Nx HPAIVs, mainly H5N2 and H5N8, have caused outbreaks in the poultry industry of Taiwan since January 2015 (*9*). In 2017, clade 2.3.4.4A H5Nx and 2.3.4.4C H5N6 HPAIVs were detected in domestic poultry. The endemic situation and continued evolution of various reassortant HPAIVs in domestic poultry warrants concern about further reassortment. Enhanced active surveillance in domestic and wild waterfowl is required to monitor the spread and onward reassortment in Taiwan and to inform the design of improved prevention and control strategies.

Technical Appendix 1 Materials, methods, and supplementary data on reassortant H5N6 clade 2.3.4.4 of highly pathogenic avian influenza airus, Taiwan, 2017.

Technical Appendix 2 Authors and originating and submitting laboratories of the sequences from the GISAID EpiFlu Database, on which this study is based.
