# Supplementary material for: Reassortant Clade 2.3.4.4 of Highly Pathogenic Avian Influenza A(H5N6) Virus, Taiwan, 2017
Source: Emerg Infect Dis. 2018 Jun;24(6):1147–9. doi: 10.3201/eid2406.172071 (PMC6004838; doi:10.3201/eid2406.172071)
Supplement: Technical Appendix 1 — Materials, methods, and supplementary data on reassortant H5N6 clade 2.3.4.4 of highly pathogenic avian influenza airus, Taiwan, 2017. [file 17-2071-Techapp-s1.pdf]

Article DOI: https://doi.org/10.3201/eid2406.172071

Reassortant Clade 2.3.4.4 of Highly Pathogenic Avian Influenza A(H5N6) Virus, Taiwan, 2017

Technical Appendix

Materials and Methods

The Dk/Tw/17 virus was detected and confirmed by egg inoculation and RT-PCR as described previously (*1*). The intravenous pathogenicity index (IVPI) test was conducted according to the OIE Manual of Diagnostic Tests and Vaccines for Terrestrial Animals (http://www.oie.int/en/international-standard-setting/terrestrial-manual). Full-length genome sequencing was performed by RT-PCR and Sanger sequencing (*1*). Nucleotide sequences have been deposited in GISAID EpiFlu under nos. EPI915867–EPI915874. For phylogenetic analysis, all available sequences of H5N6 HPAIV identified in 2016–2017 were retrieved from the EpiFlu Database on November 29, 2017. A total of 177 nt sequences for each gene were aligned using MAFFT and manual editing of alignments were performed in Geneious 8 software. The ML tree was estimated by RAxML (*2*) using the general time-reversible nucleotide substitution model. Bootstrap support values were generated by using 500 rapid bootstrap replicates. The ML phylogenetic tree was visualized with MEGA 7 software (http://www.megasoftware.net). Bootstrap values >70% are shown at the branch nodes. A median-joining (MJ) phylogenetic network of HA gene was constructed using the NETWORK ver. 5.0 (*3*).

References

1. Lee MS, Chen LH, Chen YP, Liu YP, Li WC, Lin YL, et al. Highly pathogenic avian influenza viruses H5N2, H5N3, and H5N8 in Taiwan in 2015. Vet Microbiol. 2016;187:50–7. PubMed http://dx.doi.org/10.1016/j.vetmic.2016.03.012

2. Stamatakis A. RAxML version 8: a tool for phylogenetic analysis and post-analysis of large phylogenies. Bioinformatics. 2014;30:1312–3. PubMed http://dx.doi.org/10.1093/bioinformatics/btu033

3. Bandelt HJ, Forster P, Röhl A. Median-joining networks for inferring intraspecific phylogenies. Mol Biol Evol. 1999;16:37–48. PubMed http://dx.doi.org/10.1093/oxfordjournals.molbev.a026036

Technical Appendix 1 Table. Genetic homologies of the A/duck/Taiwan/1702004/2017(H5N6) isolate to H5N6 HPAIV from Japan and South Korea

	Nucleotide identity, %		
Gene*	A/whooper swan/Korea/Gangjin 49 1/2016 collected on 2016 Nov 20	A/spot billed duck/Korea/WB141/2016 collected on 2016 Nov 10	A/teal/Tottori/2/2016(H5N6) collected on 2016 Nov 15
PB2	99.6	99.6	99.3
PB1	99.7	99.7	99.5
PA	99.7	99.7	99.4
HA	99.7	99.5	99.5
NP	99.6	99.7	99.6
NA	99.7	99.6	99.5
MP	99.8	99.8	99.6
NS	99.9	99.8	99.9

*HA, hemagglutinin; MP, matrix; NA, neuraminidase; NP, nucleoprotein; NS, nonstructural; PA, polymerase acidic; PB1 and 2, polymerase basic 1 and 2.

Detection of clade 2.3.4.4c H5N6 HPAIV in Taiwan, 2017

Date	Type of bird	No of affected farms[A]	Location (County)
2 Feb 2017	Domestic duck	A dead bird on a country road	HUALIEN
9 Feb 2017	Turkey	1	TAINAN
10 Feb 2017	Domestic duck	1	HUALIEN
13 Feb 2017	Native chicken	1	YUNLIN
	Chicken	1	CHIAYI
14 Feb 2017	Chicken	2	CHIAYI
	Chicken	1	CHIAYI
15 Feb 2017	Native chicken	1	YUNLIN
	Goose	1	CHIAYI
18 Feb 2017	Domestic duck	2	HUALIEN
6 Mar 2017	Domestic duck	1	HUALIEN

[A] Confirmed by RT-PCR and Sanger sequencing as previously described (8).

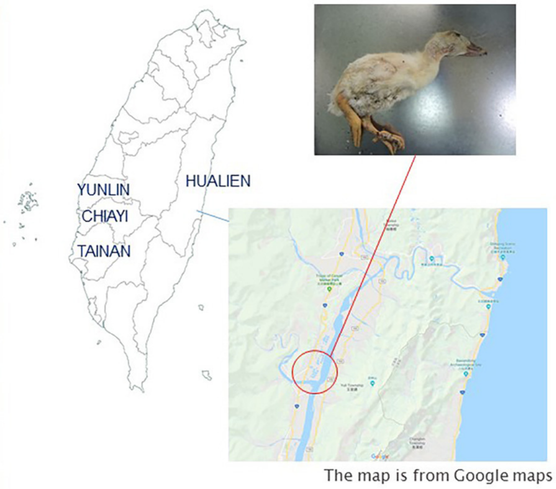

Technical Appendix 1 Figure 1. Detection of clade 2.3.4.4C H5N6 HPAIV in Taiwan, 2017.

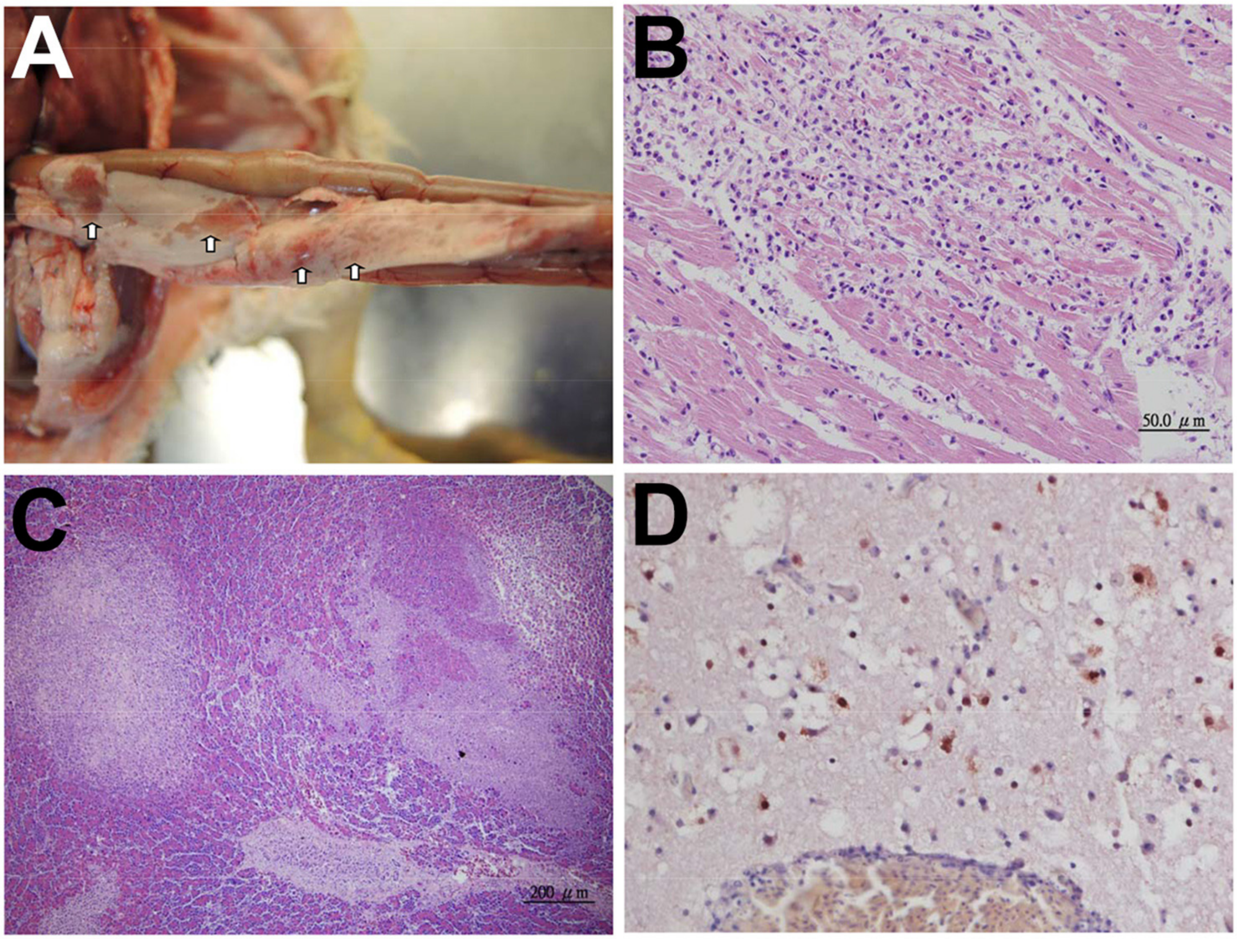

Technical Appendix 1 Figure 2. Necropsy and histopathological findings. A) Multifocal necrosis with hemorrhage in the pancreas (arrow). B) Multifocal coagulative necrosis of the pancreas. C) Heart contains widespread myocyte necrosis with prominent lymphocyte and some heterophil infiltration. D) Mild non-suppurative encephalitis evident as lymphocytic cuffing and endothelial cell hypertrophy and hyperplasia. Viral antigen is localized to neurons.

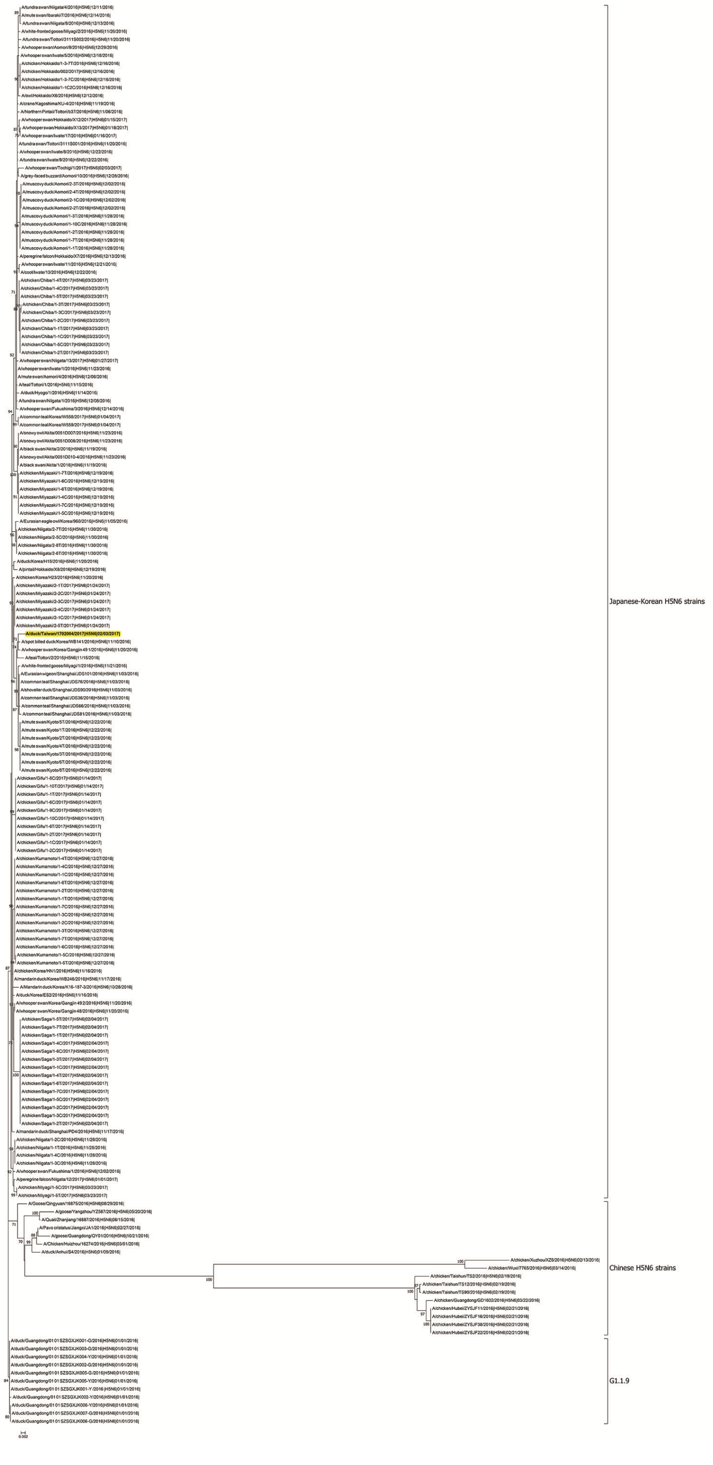

Technical Appendix 1 Figure 3. Maximum-likelihood phylogeny of the PB2 gene of H5N6 viruses identified from East Asia during 2016–2017. The percentages of replicate trees (>70%) in which the associated taxa clustered together in the bootstrap test (500 replicates) are shown next to the branches. The A/duck/Taiwan/1702004/2017(H5N6) virus is highlighted. Scale bar indicates nucleotide substitutions per site.

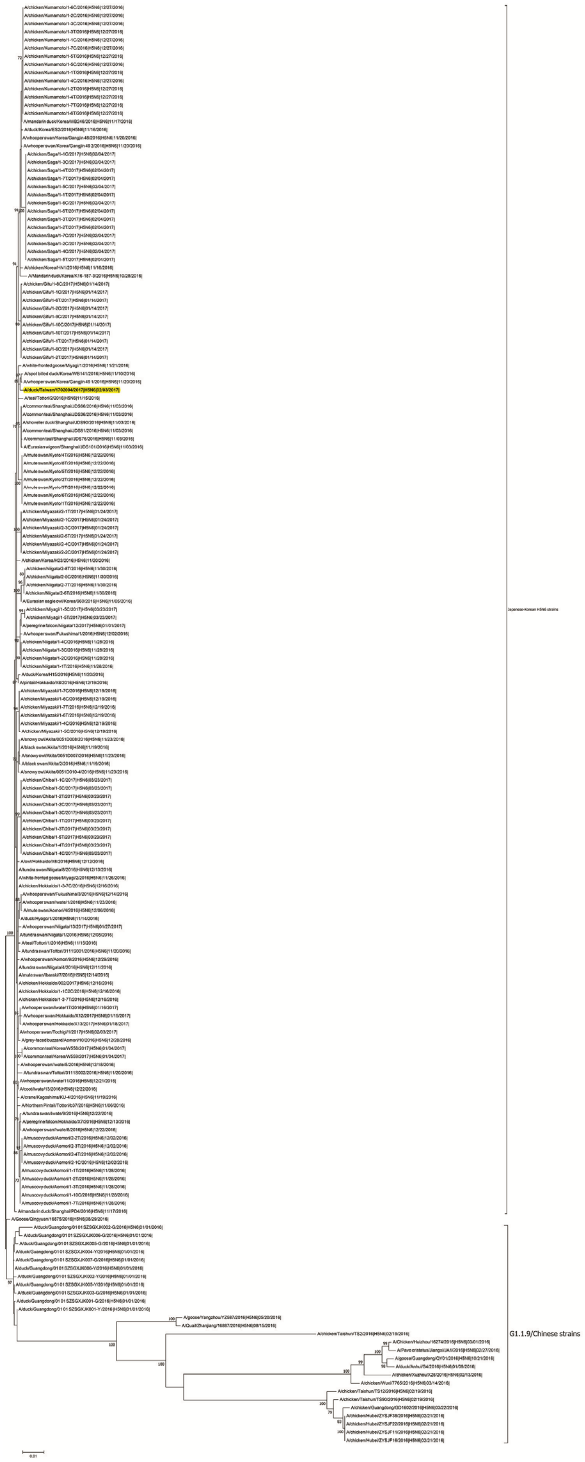

Technical Appendix 1 Figure 4. Maximum-likelihood phylogeny of the PB1 gene of H5N6 viruses identified from East Asia during 2016–2017. The percentages of replicate trees (>70%) in which the associated taxa clustered together in the bootstrap test (500 replicates) are shown next to the branches. The A/duck/Taiwan/1702004/2017(H5N6) virus is highlighted. Scale bar indicates nucleotide substitutions per site.

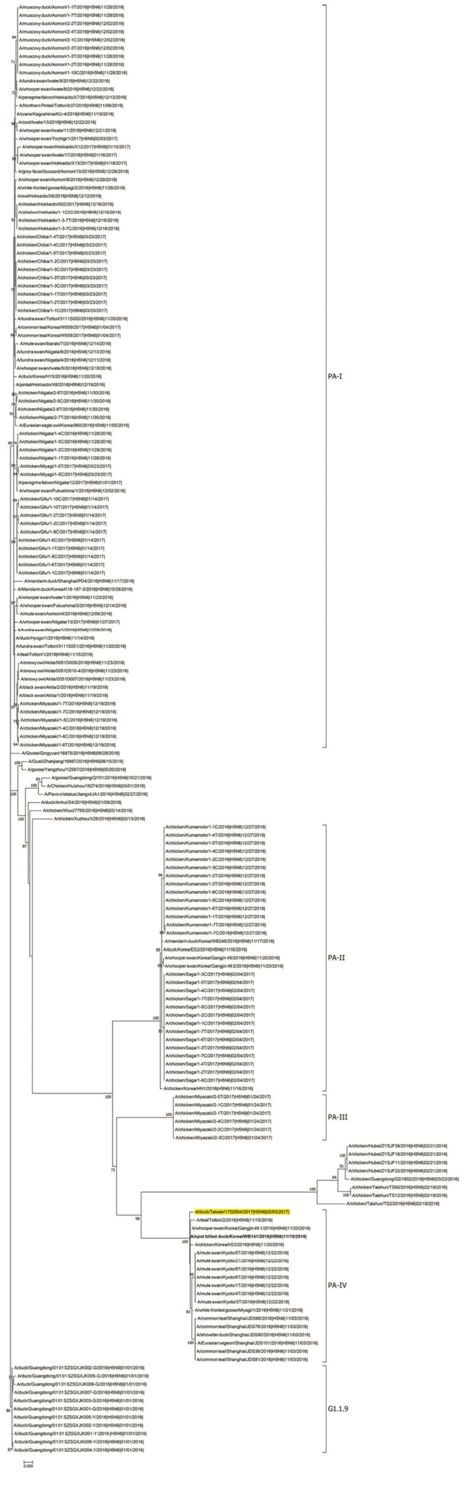

Technical Appendix 1 Figure 5. Maximum-likelihood phylogeny of the PA gene of H5N6 viruses identified from East Asia during 2016–2017. The percentages of replicate trees (>70%) in which the associated taxa clustered together in the bootstrap test (500 replicates) are shown next to the branches. The A/duck/Taiwan/1702004/2017(H5N6) virus is highlighted. Scale bar indicates nucleotide substitutions per site.

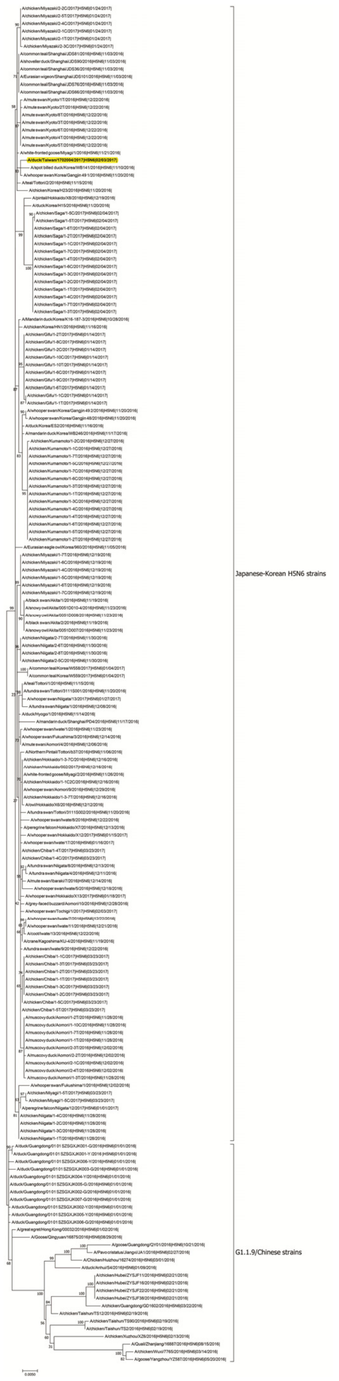

Technical Appendix 1 Figure 6. Maximum-likelihood phylogeny of the HA gene of H5N6 viruses identified from East Asia during 2016–2017. The percentages of replicate trees (>70%) in which the associated taxa clustered together in the bootstrap test (500 replicates) are shown next to the branches. The A/duck/Taiwan/1702004/2017(H5N6) virus is highlighted. Scale bar indicates nucleotide substitutions per site.

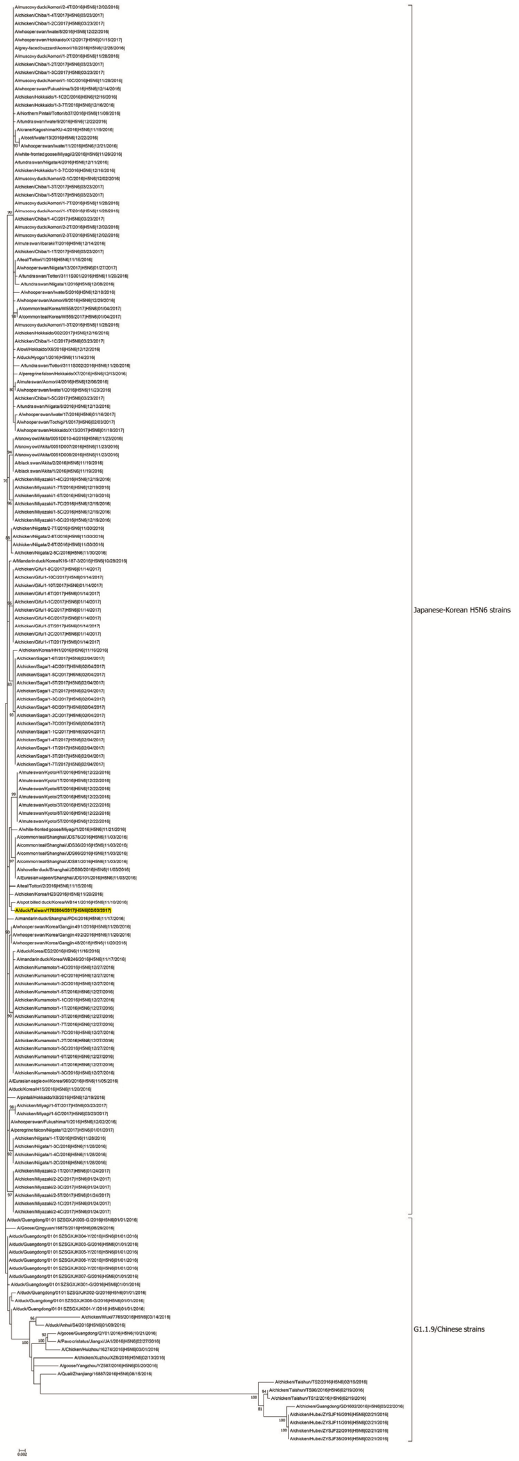

Technical Appendix 1 Figure 7. Maximum-likelihood phylogeny of the NP gene of H5N6 viruses identified from East Asia during 2016–2017. The percentages of replicate trees (>70%) in which the associated taxa clustered together in the bootstrap test (500 replicates) are shown next to the branches. The A/duck/Taiwan/1702004/2017(H5N6) virus is highlighted. Scale bar indicates nucleotide substitutions per site.

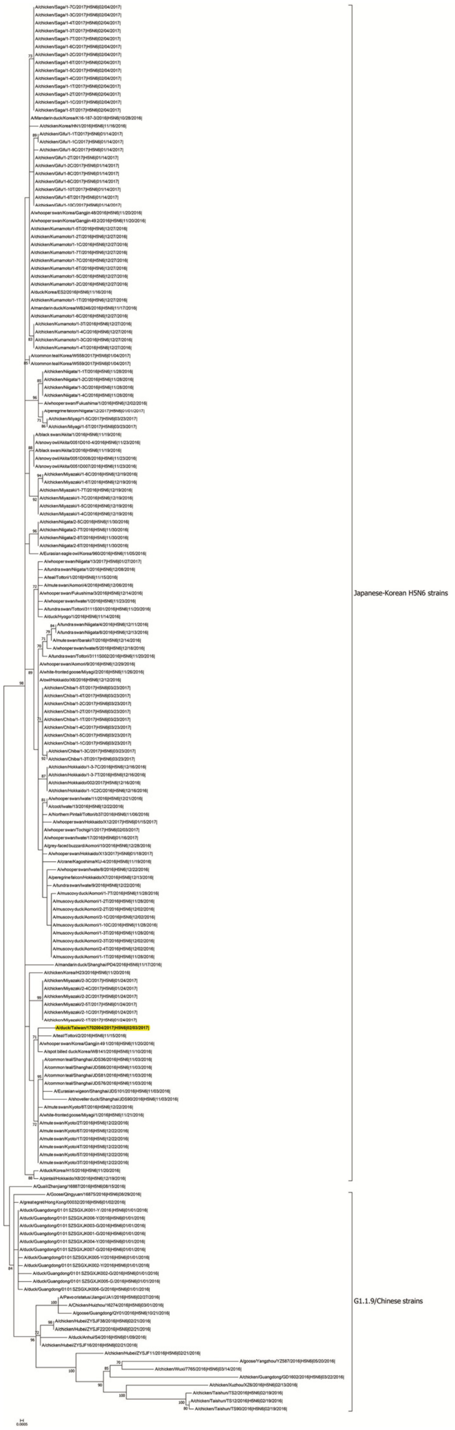

Technical Appendix 1 Figure 8. Maximum-likelihood phylogeny of the NA gene of H5N6 viruses identified from East Asia during 2016–2017. The percentages of replicate trees (>70%) in which the associated taxa clustered together in the bootstrap test (500 replicates) are shown next to the branches. The A/duck/Taiwan/1702004/2017(H5N6) virus is highlighted. Scale bar indicates nucleotide substitutions per site.

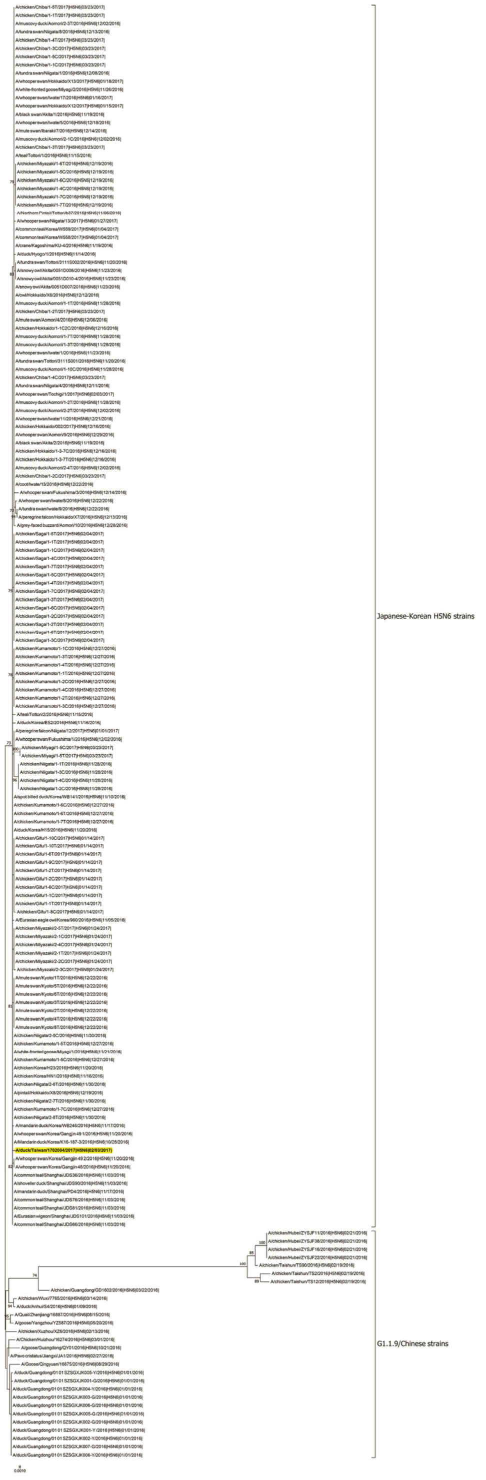

Technical Appendix 1 Figure 9. Maximum-likelihood phylogeny of the M gene of H5N6 viruses identified from East Asia during 2016–2017. The percentages of replicate trees (>70%) in which the associated taxa clustered together in the bootstrap test (500 replicates) are shown next to the branches. The A/duck/Taiwan/1702004/2017(H5N6) virus is highlighted. Scale bar indicates nucleotide substitutions per site.

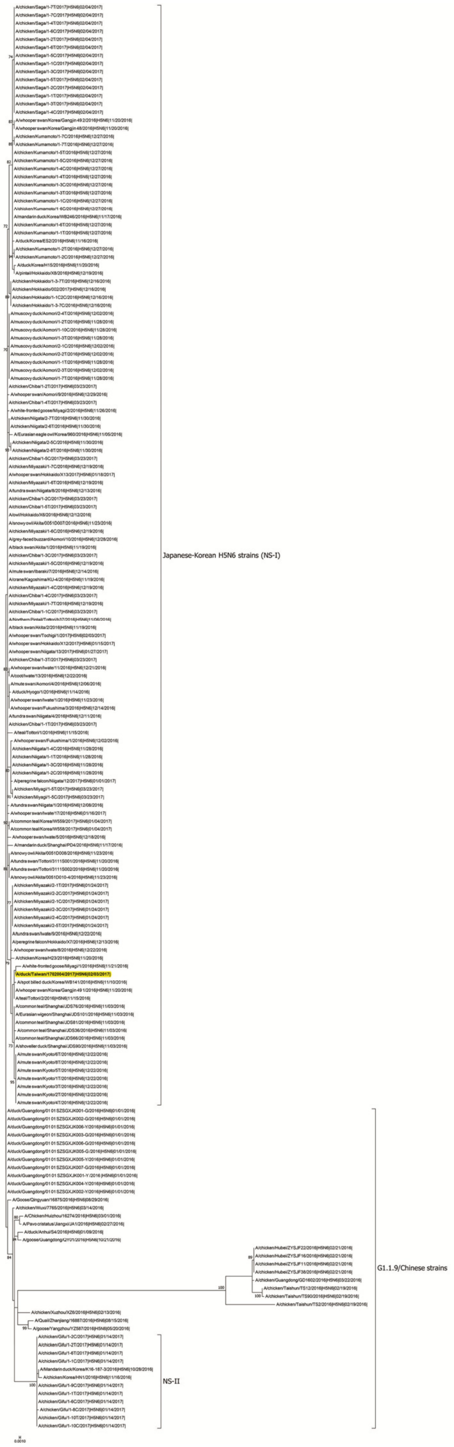

Technical Appendix 1 Figure 10. Maximum-likelihood phylogeny of the NS gene of H5N6 viruses identified from East Asia during 2016–2017. The percentages of replicate trees (>70%) in which the associated taxa clustered together in the bootstrap test (500 replicates) are shown next to the branches. The A/duck/Taiwan/1702004/2017(H5N6) virus is highlighted. Scale bar indicates nucleotide substitutions per site.
